# Secondary infertility caused by the retention of fetal bones after an abortion: a case report

**DOI:** 10.1186/1752-1947-2-208

**Published:** 2008-06-17

**Authors:** Hannah MC Kramer, Johann PT Rhemrev

**Affiliations:** 1 Medisch Centrum Haaglanden, Department of Gynaecology, Delft, The Netherlands; 2 Bronovo Ziekenhuis, Department of Gynaecology, The Hague, The Netherlands

## Abstract

**Introduction:**

Unwanted contraception through prolonged retention of fetal bone is a rare cause of secondary infertility. It is usually associated with a history of abortion, either spontaneous or induced.

**Case presentation:**

We describe a case of intrauterine retention of fetal bone diagnosed 8 years after the termination of a pregnancy. The patient had no complaints of pain, irregular vaginal bleeding or discharge. A hysteroscopy was performed and irregular structures were removed. These fragments were fetal bones, which probably functioned as an intrauterine contraceptive device. After removal of the fetal bone fragments the patient conceived spontaneously within 6 months.

**Conclusion:**

This case report stresses the importance of taking a thorough history and evaluation of the endometrium by transvaginal ultrasound or hysteroscopy in women with secondary infertility.

## Introduction

Since 1973 over 38 million legal abortions have been performed in the USA. Approximately 90% of these abortions were performed on patients between 15 and 34 years of age [1].

All recent and large-scale studies show that legally performed abortions were extremely safe procedures with a lethal risk rate of 0 to 0.7 per 100,000. The overall early complication rate due to hemorrhage, uterine perforation or cervical injury is between 0.01% and 1.16% [2]. So far, definite conclusions cannot be made as to the impact of multiple induced abortions and reproductive problems. Complications due to previous abortions such as Asherman's syndrome may occur. Since 50% of abortions are performed on young patients, these women may develop future fertility problems caused by previous procedures.

We describe a rare cause of secondary infertility due to prolonged retention of intrauterine bone after a dilatation and curettage. A number of case reports have been published on the prolonged retention of fetal bones up to 23 years after an abortion, either spontaneous or induced [3]. Most patients complain of dysmenorrhea, dysfunctional uterine bleeding, pelvic pain, dyspareunia, vaginal discharge or spontaneous passage of fetal bones [4]. Cases are discovered by vaginal ultrasound examination [5,6], hysterosalpingography [7] and, in particular, hysteroscopy [8]. In our case, the patient had no complaints other than secondary infertility.

## Case presentation

A 32-year-old woman presented with secondary infertility, her only pregnancy being a termination 8 years previously at 12 weeks' gestation in Nigeria. Fertility work-up showed a regular menstrual cycle, with no biphasic temperature curve. The semen of the male partner was normospermic.

**Figure 1:**
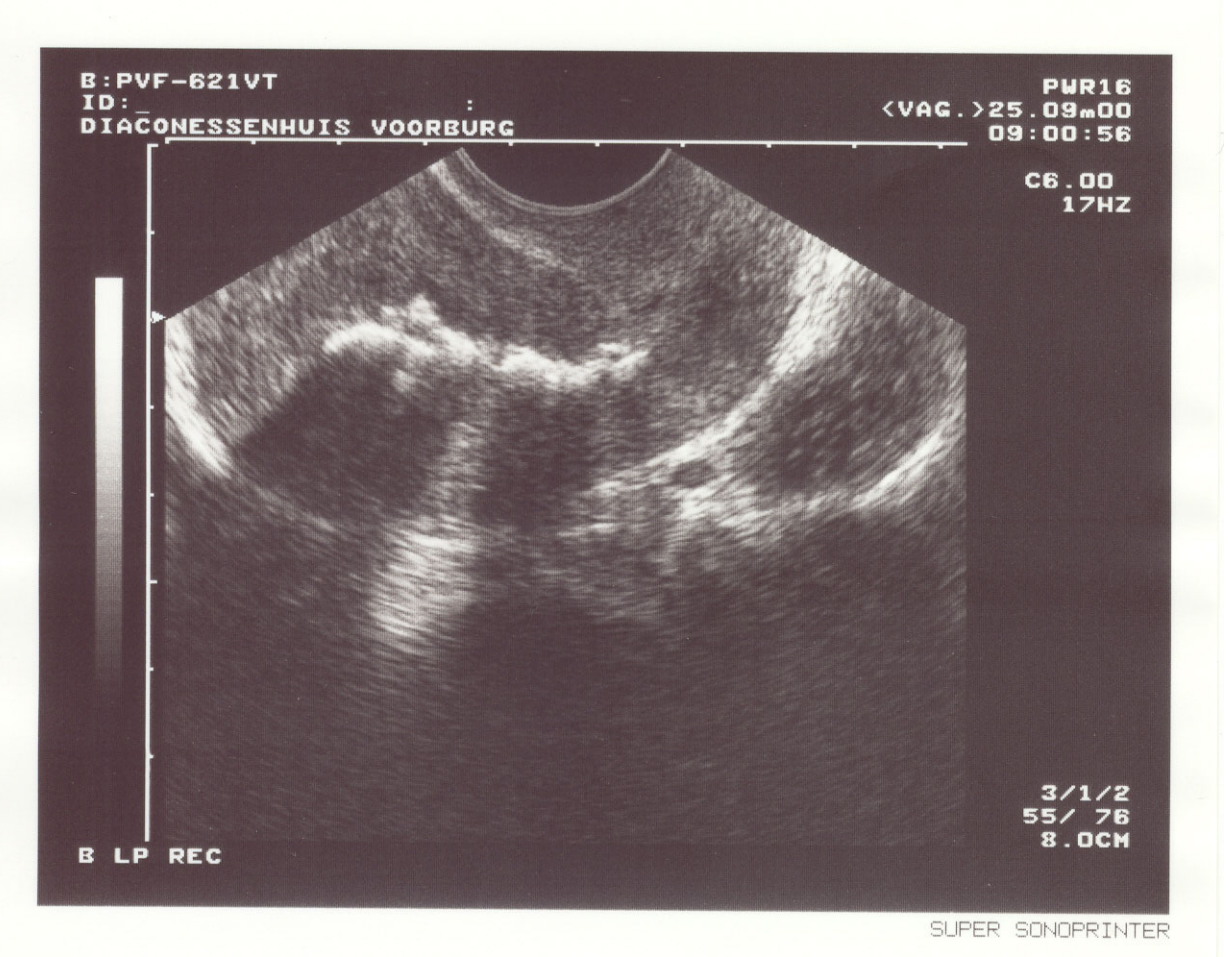
Scarred endometrium revealed on transvaginal ultrasound.

**Figure 2:**
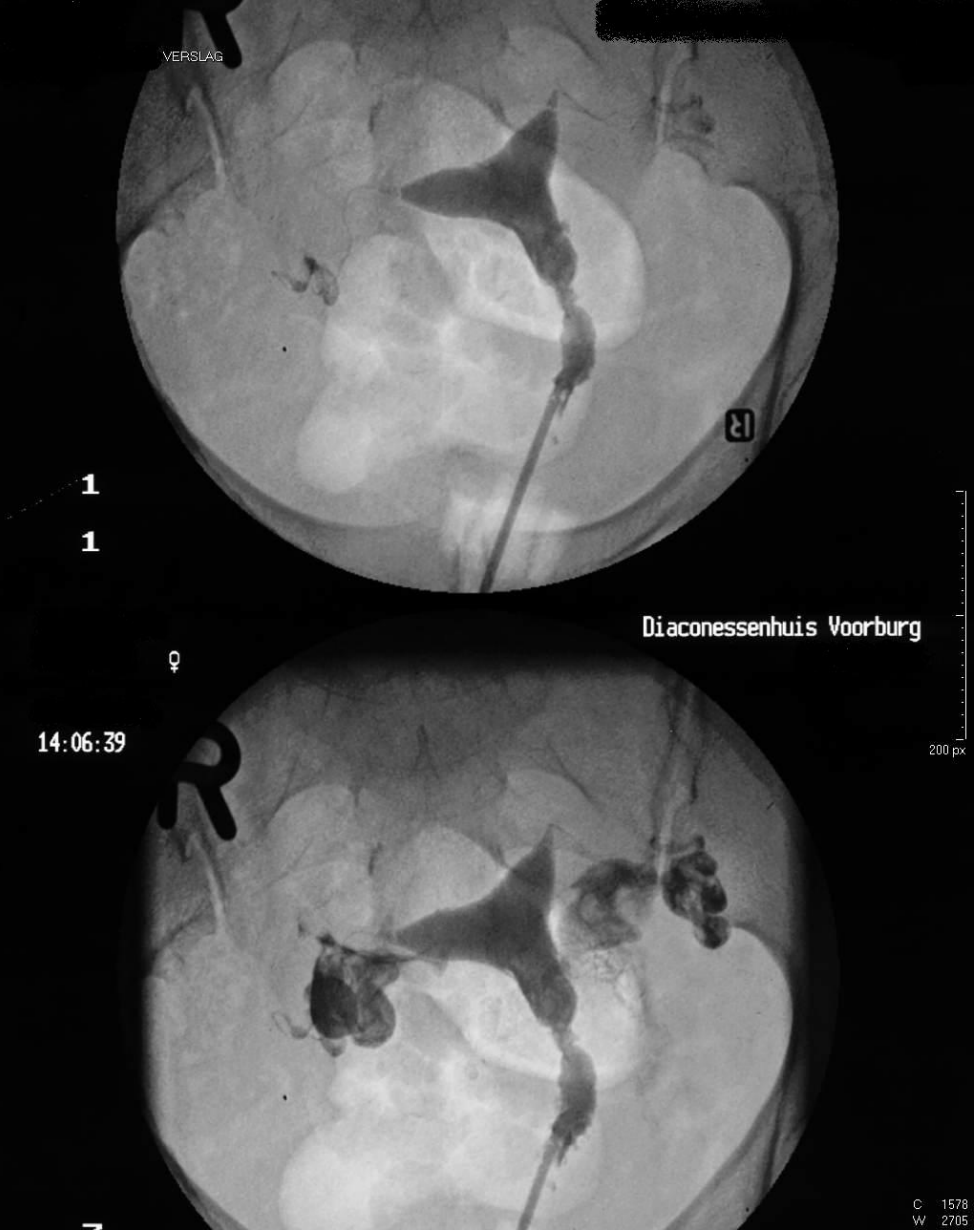
Hysterosalpingogram of a normal uterus.

A transvaginal ultrasound revealed a normal-sized, normal-shaped uterus without myoma with an echogenic scarred endometrium (Figure 1). A laparoscopy showed no signs of pathology of the fallopian tubes or Fitz-Hugh-Curtis syndrome. During a hysteroscopy, an Asherman's syndrome was evident, which was treated with adhesiolysis and an intrauterine contraceptive device (IUCD) for a period of 3 months. After removal of the IUCD, a hysterosalpingogram showed no abnormalities (Figure 2).

**Figure 3:**
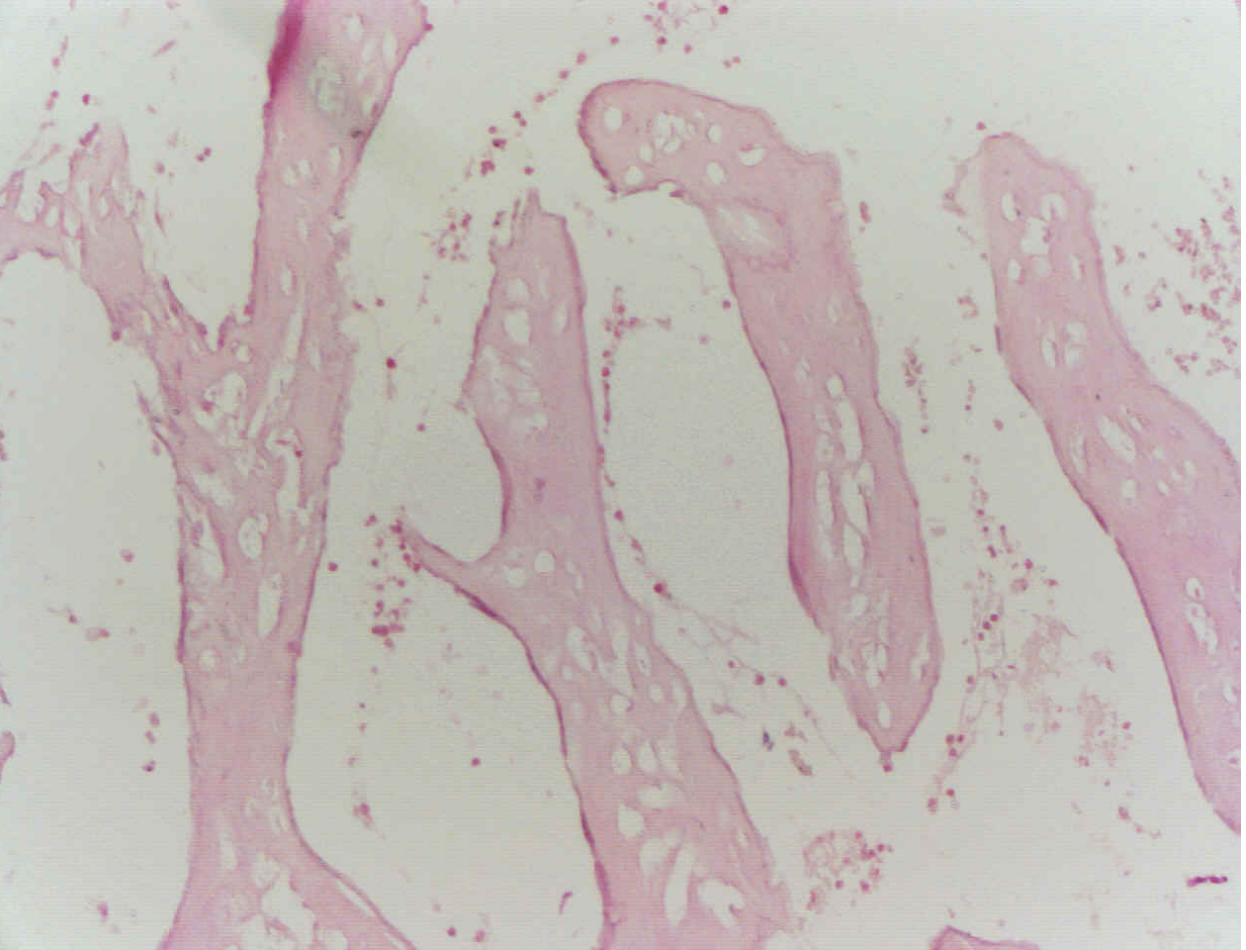
Histopathological confirmation of the presence of fetal bones.

The patient was treated with six cycles of clomiphene citrate which, despite ovulatory menstrual cycles, did not result in a pregnancy. Thereafter, three *in vitro *fertilization (IVF) cycles were performed in which qualitative perfect blastocysts were transferred without conception. The cause of this problem was thought to be malfunctioning endometrium. A second hysteroscopy was performed during which irregular structures, suspected to be bone fragments, were removed. Histopathological analysis confirmed that the structures contained five bone fragments (Figure 3). Two months later, a second hysteroscopy showed a normal empty uterine cavity with a normal endometrial lining.

Two months after the last hysteroscopy, while waiting for the IVF to commence, the patient became pregnant spontaneously with twins. Two healthy children were delivered. Two years later she delivered another healthy child.

## Discussion

Every year many abortions are performed. Although abortion is an extremely safe procedure, complications do occur. This case illustrates a rare cause of secondary infertility due to the prolonged retention of fetal bones where the patient had no complaints of pain, irregular vaginal bleeding or discharge. Furthermore, this case shows fertility can be increased following removal of the fetal bones: within 6 months of the hysteroscopy and removal of the bone fragments, the patient became pregnant spontaneously. As in other case reports, this suggests that the presence of fetal bone in the uterine cavity may act as an IUCD [3-7]. In [3], a spontaneous pregnancy was possible following the removal of 15-year-old retained fetal bones.

The diagnosis of retained fetal bone can be made by identifying a filling defect on a hysterosalpingogram, an echogenic area on vaginal ultrasound or, as in our case, by direct visualization on hysteroscopy.

When the bone fragments are deeper in the endometrium they can be overlooked by hysteroscopy and the only indication may be an echogenic area in the endometrium on transvaginal ultrasound [5].

## Conclusion

We recommend a transvaginal ultrasound or hysteroscopy on every patient with a history of secondary infertility following abortion, regardless of the interval between the preceding termination and presentation. Taking a thorough history is a prerequisite. A high success rate may be expected following the removal of any retained bones.

## Abbreviations

IUCD: intrauterine contraceptive device; IVF: *in vitro *fertilization.

## Competing interests

The authors declare that they have no competing interests.

## Consent

Written informed consent was obtained from the patient for publication of this case report and any accompanying images. A copy of the written consent is available for review by the Editor-in-Chief of this journal.

## Authors' contributions

HMCK provided the background data and wrote the manuscript, JPTR provided the patient information, and was involved in drafting the manuscript and providing feedback on earlier drafts. Both authors approved the final manuscript.
